# Prediction and Analysis of Contemporary College Students' Mental Health Based on Neural Network

**DOI:** 10.1155/2022/7284197

**Published:** 2022-07-13

**Authors:** Jingjing Pei

**Affiliations:** School of Education, Xi'an Fanyi University, Xi'an, Shannxi 710105, China

## Abstract

For an ordinary student who has just graduated from high school, interpersonal communication and performance evaluation on campus is also a huge challenge. In order to solve the future and current competition and pressure faced by contemporary college students, many college students have mental health problems. This paper evaluates, predicts, and analyzes the mental health status of contemporary college students based on a neural network algorithm. The computer technology of neural network algorithm is applied to the prediction of contemporary college students' mental health. Data mining technology based on a neural network algorithm is used to collect data sources. Finally, the prediction results are analyzed, and the main psychological stressor factors of contemporary college students are analyzed by cluster analysis. The results show that there is no significant correlation between college Students' inferiority complex and dependency map and the incidence of mental diseases and majors. A comprehensive physical symptom test was conducted on individuals to understand students' psychological characteristics and behavior.

## 1. Introduction

Education is the foundation of the centennial plan. If China wants to realize the historical task of the great rejuvenation of the Chinese nation, it is inseparable from education. China has implemented basic education for 70 years, which is also the true embodiment of China's transformation from backwardness to rejuvenation [1]. When new China was founded in 1949, China prospered with all kinds of waste, and the average time of education was only 1.6 years. By 2018, with the popularization of nine-year compulsory education in China, the average duration of education has increased to 10.6 years [2]. The improvement of per capita education time also reflects the improvement of the overall quality of our citizens and the enhancement of the overall strength of our country. As the saying goes, the country prospers when the youth prospers; a strong youth makes a strong country. To achieve the great goal of building a powerful socialist country, it is inseparable from the overall improvement of national quality brought by education.

With the gradual increase of China's attention to education, the number of higher talents in China has also increased rapidly, and the number of college students has increased by more than 40 times [3]. Newly enrolled college students have just broken free from the “cage” of the college entrance examination. In the face of the new environment of college life, they often have a psychological gap. Combined with some other comprehensive reasons, they cause college students' psychological problems. First of all, for example, there is a big gap between college life and high school life. Some students are prone to maladjustment in the face of the new university learning environment, learning methods, and teachers and students. If this maladjustment problem is not reasonably adjusted, it will gradually develop into psychological problems [4]; Secondly, self cognitive disorder is also a common psychological problem among college students. Students admitted to the same university usually have a small gap in overall level and college entrance examination scores, but some students may have outstanding grades in class and even grade in high school. Then, when students suddenly enter an environment with excellent students around them, they may have concerns about poor academic performance or some other deviations in self cognition, which may lead to relevant psychological problems [5]; In addition, interpersonal relationship is also an important part of college life. In middle school life, everyone's main task is to study. Usually, in addition to class, they do not have too much time for private communication. However, in college, we often face the same dormitory or teachers and classmates who meet and communicate frequently, which will produce relatively complex interpersonal relationships compared with middle school. At this time, some students may be relatively isolated or lack corresponding communication skills, resulting in poor interpersonal relations, even some conflicts, resulting in isolation, and finally develop into interpersonal barrier problems. In addition, emotional instability and poor adaptability caused by factors such as adolescence are also common psychological problems of college students [6].

In recent years, the psychological problems of college students have attracted more and more attention. Relevant studies have shown that college students' psychological problems have become one of the important reasons for college students' dropping out, weariness, self mutilation, and suicide, and the mental health status of college students is generally lower than that of their peers [7]. The mental health of college students is not only related to the impact of their completion of college education but also related to the impact of students entering society after graduation. College life is the prelude for individuals to step into social life. If a student has a certain psychological problem in college life, this psychological problem may accompany his whole social life in the future, and even produce various other psychological problems in his future social life. Therefore, the Chinese government began to protect students' mental health very early. In 1994 and 2010, China clearly proposed to carry out education and counseling on students' mental health at different levels, in a variety of methods and angles in the opinions of the CPC Central Committee on further strengthening and improving school moral education and in the outline of the national medium and long-term education reform and development plan for 2010–2020, at the same time, it is clear that we should pay attention to students' psychological education, promote students' mental health, shape students' strong physique and temper students' strong will while teaching knowledge. These are to cultivate children's positive psychology from urination and avoid the probability of psychological problems in the future [8].

In addition to the prevention and counseling of common psychological problems of college students in advance, we should pay more attention to the mental health problems of college students. However, due to China's historical factors, patients with psychological problems usually do not take the initiative to choose to see a doctor but think that it is better under the change of thought, which often leads to irreversible psychological problems. Therefore, timely discovering and intervening in the mental health status of contemporary college students is the best way to avoid serious mental problems. We hope that through the algorithm of the neural network, we can analyze the mental health status of college students, timely find students with abnormal mental states and predict their future mental state, so as to prevent accidents.

## 2. Related Work

For the study of College Students' mental health, relevant psychological crisis theories have been put forward for a long time. The earliest theory of psychological crisis was put forward in 1944. After 20 years, psychologists perfected and developed the published psychological crisis theory, formed a more systematic psychological crisis theory, and established a college students' psychological crisis model on this basis [9]. Later, when advocating the theory of psychological crisis, he put forward the concepts of mental health and mental health and believed that mental health was more important than a psychological crisis. Modern scholars believe that with the development of network media, under the background of the complex network environment, the psychological pressure of college students may be adversely affected, thus affecting the psychological problems of college students [10]. The researchers tested the psychological state of college students who used social media for a long time and found that college students who relied too much on social media generally had a higher degree of depression than their peers. Their ability to adapt to the environment and learning disabilities are also relatively lower than their peers, and the incidence rate of mental diseases is also significantly increased. This paper reviews the mental health status of college students. At present, it is generally believed that the poor mental health of college students can be effectively improved through cognitive behavior and other related treatment [11]. Since the 1990s, China's psychological education has begun to flourish. During this period, many psychologists began to pay attention to the relevant theories of psychological crisis and applied them to the study of College Students' mental health, and achieved some results. Through the analysis of College Students' family environment, it is found that family factors have a certain impact on College Students' psychological state, that is, the impact on the original family. Under the influence of the original family, this paper further discusses the influence of personal character and the psychological status of college students during college and later into society. This paper expounds in detail on the influence and relationship of four factors of character learning school, local family, and society on College Students' mental health [12]. Usually, we classify personal factors as internal factors affecting college students' mental health, including personal genetic factors, physiological factors, and psychological factors. School, native family, and society are external factors. External factors affect college students' mental health to a certain extent, but internal factors are the determinants of their mental health. However, in a specific environmental context, external factors may also play a relatively decisive role.

In addition to the relevant theoretical research on the mental health status of college students, the commonly used research methods for the mental health status of college students mainly include the investigation method, cause analysis method, and scale method. At present, the commonly used mental health test scale for college students in China is the self-assessment scale SCL-90. Through the statistics of students' measurement results, then carry out the correlation analysis of significant differences, do the binary logistic regression, and finally get the correlation between psychological status and some factors. The optimal value can also be solved by a linear regression equation to obtain the value of an influencing factor when the psychological condition maintains the optimal level [13]. Through binary logistic regression, sun Chunyang and others finally found that the most important factors affecting college students' mental health are compulsion, interpersonal relationships, and depression. According to the research of Wang Chun and others, 49.55% of college students have related psychological problems or psychological pressure, among which family economic level and parental education are one of the main causes of psychological problems or psychological pressure [14]. In addition, Chinese scholars also studied the effects of gender, grade, parents' marital satisfaction, employment pressure, nationality, place of origin, and other factors on College Students' mental health. The final results show that the mental health status of contemporary college students is affected by various factors such as family, school, and society, and there is also a high correlation with their personal living habits, personal personality, and related interpersonal relationships [15, 16].

To sum up, the research on the mental health status of contemporary college students has achieved rich results. However, at present, the research on the mental health status of contemporary college students is mainly through single factor and multifactor analysis, and the way of data collection is mainly through questionnaire survey. The data collected by this method has certain limitations, and there is a certain observer effect when the subjects fill in the data, so the authenticity of the data needs to be further verified. This paper attempts to carry out data mining through a neural network algorithm. The data mined can most truly and intuitively reflect the psychological state of the subjects. Then, predict and evaluate through a certain model algorithm and finally achieve accurate prediction and analysis. In the case of poor mental health of college students, carry out early intervention and counseling to avoid the adverse consequences caused by psychological problems.

## 3. Method

**Figure 1:**
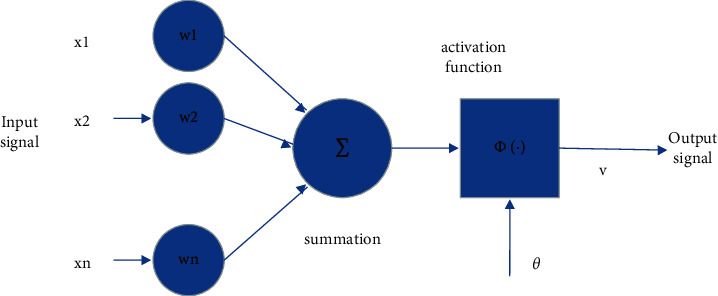
Basic neuron model.

The algorithm of modern neural network evolved from a large amount of computing work of computer. With the application of computer in computing work, people gradually find that computer work is better in solving a large number of complex calculations, but it is only suitable for information processing or mathematical computing. However, there are often some errors in dealing with some logical problems such as judgment and decision-making or pattern recognition. In the final analysis, it is because people often need to consider the complex environment and objective conditions when making some logical calculations such as decision-making. However, because the computer can only execute the 01 code instructions compiled in advance, it can not combine the objective conditions or the optimal conclusion expected by people, nor can it simulate human subjective emotion. At the same time, because the computer can not solve or consider multiple aspects in a decision logic problem at the same time, it can only carry out linear calculations step by step. The human brain is interconnected by multiple neurons. Each neuron can receive or transmit release signals at the same time. When dealing with decision logic problems, parallel operations can also be carried out. On the whole, for decision logic problems, the neural structure of the human brain is more efficient than that of a computer. Computer related researchers gradually come up with the algorithm model of computer neural network according to the biological characteristics of the brain. Therefore, the essence of a computer neural network is to use a computer to simulate the operation mode of the brain neural network for information processing. This information processing can achieve the optimal result in the complex calculation process. At the same time, the structure of the connection between each link can also greatly improve the calculation efficiency. Therefore, computer neural network structure is a computer model widely used in practical problem solving in recent years. The computer neural network model is mainly composed of three layers of networks, which are called input layer, hidden layer, and output layer, respectively. These three-layer networks constitute the basic structure of a computer neural network and a complete neural link in the brain. The input layer controls the input neurons in a complete nerve conduction, the output layer controls the output neurons in the nerve conduction, and the hidden layer undertakes the signal transmission between the input and output neurons and the complex operation of output command decision-making. In model calculation, we usually give an initial value in the input layer. Then, the parameters and training rules are set in advance in the hidden layer, which is divided into full connection and linear connection according to the type of neural network. Different connection modes compare different training modes in the hidden layer. The fully connected hidden layer is that the neurons in the hidden layer can transmit results and train each other; The linear connection is that the training results are transmitted linearly in the hidden layer, which can only be transmitted from the previous neuron to the next neuron but can not be transmitted reversely or one to many neurons in the same layer. The training results are trained in the hidden layer and transmitted to the output layer. The output layer outputs the final results. Although the structure of the neural network is mainly divided into three layers, each layer can contain multiple neurons, and each neuron has its own operation model. Generally speaking, each neuron is composed of three elements, and the input signal is responsible for the energization of the initial value; the summation function is to regularize the summation signals from different directions to facilitate the operation of subsequent activation functions; the activation function is compared with the biological signal processing inside a single neuron in a biological neuron, which is also the training result of the initial value in a single neuron. Finally, the training results of this neuron are output to the next neuron.  Figure 1.

After determining the basic algorithm used in the prediction and analysis of contemporary college students' mental health, we will continue to test the accuracy of the algorithm, and finally, determine an algorithm model with the highest prediction accuracy. The traditional BP neural network prediction model is one of the most widely used models at present. It has the advantages of simple operation and a small amount of calculation. However, when calculating a large number of data, the learning efficiency of the neural network is low, and the calculation speed is also slow. The neural network initializes multiple neural networks with different parameter values and takes the smallest as the result. At the same time, it is different from the accurate calculation of gradient by standard gradient descent. The random gradient descent method adds random factors in calculating the gradient, so even if it falls into a local minimum point. The calculated gradient may still not be 0, so it is possible to jump out of the local minimum and continue the search. At the same time, the BP algorithm is often easy to fall into the problem of the local optimal solution in the calculation process, so that the final solution result is only optimal in a user-defined interval but not necessarily the optimal value in the specified global problem, resulting in some adverse consequences. Therefore, this paper makes some improvements on the basis of the BP neural network and adds the graph neural network model based on the convolution neural network algorithm to predict and analyze the mental health status of contemporary college students.

**Figure 2:**
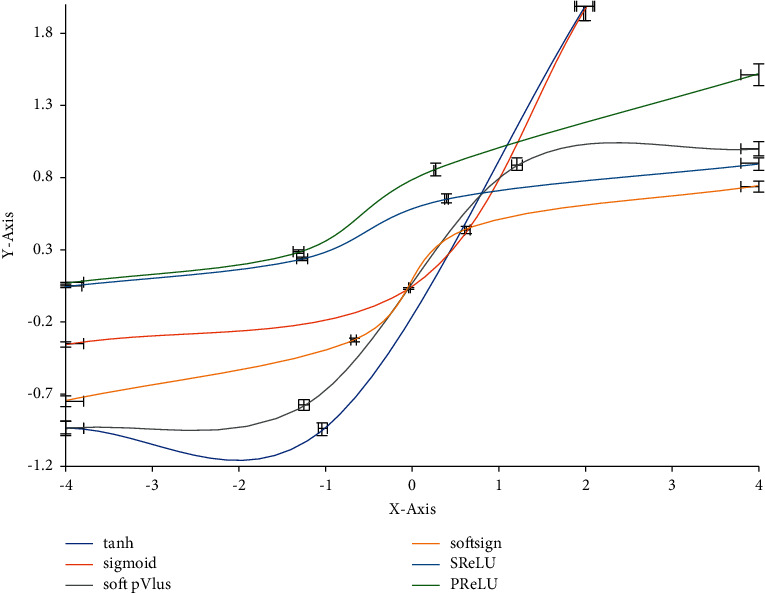
Images of different activation functions.

The basic structure of the neural network model proposed in this paper is also the input layer, output layer, and hidden layer. The input layer and output layer only play the role of transmission in the overall structure, so only the algorithm on the same single neuron as the hidden layer can be determined without an additional algorithm. The commonly used algorithms in a single neuron include linear activation function, natural exponential activation function, sigmoid activation function, and hard_ Sigmoid activation function. the activation function image is shown in Figure 2. It can be seen from the image that the change area of the sigmoid function is relatively uniform, and the sigmoid function is often used for discrete model prediction. Although its function image is nonlinear, its derivative changes linearly, so the sigmoid function increases monotonically in the definition domain. Sigmoid function expression and derivative expression are shown in the following formula:

(1) $$\begin{array}{c} \sigma \left(z\right)=\frac{1}{1+e^{-z}}. \end{array}$$

In addition, for the summation of the whole input value, we adopt the linear regression process. Linear regression is a method often used in geometric algebra to predict unknown parameter values according to known parameter values. Specific formula (2) can be seen. In specific cases, we also use vector form for the operation. The vector form is shown in formula (3).

(2) $$\begin{array}{c} f\left(x\right)=w_{1}x_{1}+w_{2}x_{2}+\cdots +w_{n}x_{n}+b, \end{array}$$

(3) $$\begin{array}{c} f\left(x\right)=w^{T}x+b. \end{array}$$

We fit the input value and then transfer it to the sigmoid function after preliminary integration. That is, the result of linear regression is brought into the sigmoid activation function to complete the formula of logical regression as shown in the following formula:

(4) $$\begin{array}{c} f\left(x\right)=\frac{1}{1+e^{-\left(w^{T}x+b\right)}}. \end{array}$$

In linear regression, we usually use the maximum likelihood method for fitting. When the difference between the overall real value and the predicted value reaches the minimum, it is considered to be the optimal model. In the complete formula of the sigmoid function brought by fitting value, we use the maximum likelihood method to obtain the best coefficient w of the complete formula. See formula (5) for the solution formula of the best coefficient *w*.

(5) $$\begin{array}{c} w=\max\prod_{i=1}^{m}p\left(\frac{y_{i}}{x_{i}}\right). \end{array}$$

In the practical solution, in order to simplify the calculation, the expression is usually reduced to the following formula:

(6) $$\begin{array}{c} w=\max\prod_{i=1}^{m}\ln\;p\left(\frac{y_{i}}{x_{i}}\right). \end{array}$$

**Figure 3:**
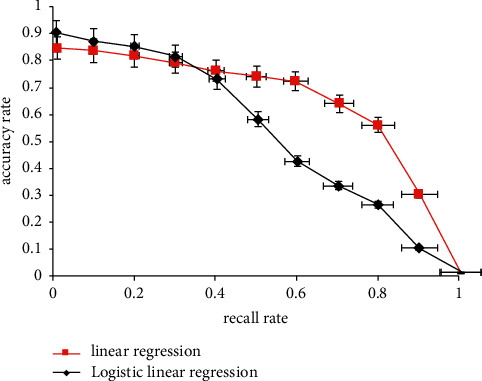
Change of accuracy between traditional linear regression and logical linear regression.

Finally, by comparing the accuracy of the traditional prediction model based on the linear regression algorithm with that based on the sigmoid function, it can be found that the accuracy of predicting the real value is higher through the action of activation function in neurons. However, under the influence of the gradual increase of recall rate, the training process of the logical neural network is relatively more complex, so it is greatly affected and the accuracy rate decreases greatly. Therefore, in practical application, we should pay extra attention to and control the influence of irrelevant variables. In the process of building the model, we need to use the validation data to test the currently built model to get the loss of the model and the accurate validation loss and accuracy. After the model is built, the model is tested with test teaching data and the accuracy is obtained. If there is a big difference between the accuracy and verification accuracy, it indicates that the model is overfitted. The specific change trend is shown in Figure 3.

It can be seen from Figure 3 that when the recall rate is 0.4, the accuracy rate of logistic linear regression is not as good as that of traditional linear regression. When the recall rate is greater than 0.4, the accuracy of traditional linear regression is not as good as that of logistic linear regression.

After determining the activation function of each basic neuron, we can determine the training method of the hidden layer. Considering that there should be many image factors in the data referred to in the prediction of college students' mental health, here we preliminarily plan to use a convolutional neural network as the parameter model of the hidden layer. A convolutional neural network is a widely used model in image analysis because of its full connection in neuron training and no timing of transmission. The transfer formula is shown in the following formula:

(7) $$\begin{array}{c} H^{l+1}=\sigma \left({\bar{D}}^{-\left(1/2\right)}AD^{-\left(1/2\right)}H^{l}W^{l}\right). \end{array}$$

**Figure 4:**
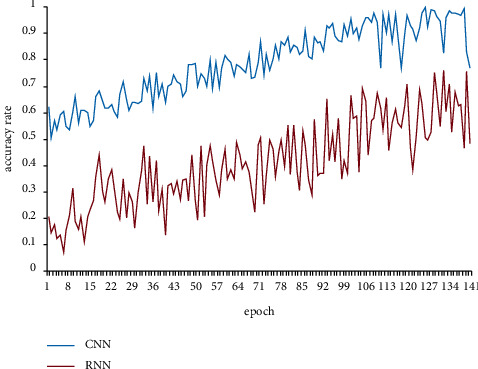
Comparison of accuracy between convolutional neural network and cyclic neural network.

Through formula (7), the parameters of the input layer are transferred to the hidden layer through the matrix, which is trained by the basic neurons of the hidden layer, and finally, a new convolution layer is formed. The new convolution layer is then transmitted downward and finally output to the output neuron. In the hidden layer, the propagation formula transmitted from the upper convolution layer to the lower layer is shown in formula (8). The accuracy of the model based on the convolution neural network and the accuracy of the cyclic neural network model are shown in Figure 4.

(8) fX,A=softmaxA^ReLuA^XW0W1.

It can be seen from Figure 4 that the fitting accuracy based on a convolutional neural network is higher, and the accuracy fluctuation is smaller with the increase of training iterations compared with the cyclic neural network. Considering that the model mentioned above is easy to be affected by irrelevant variables, we choose the convolutional neural network to further maintain the stability of the model.

Next, we avoid the influence of irrelevant variables through the error training of the hidden layer, so as to minimize the influence of irrelevant variables such as environmental variables on the accuracy of model fitting. Suppose the input value of the *i*th neuron in the hidden layer is as follows:

(9) $$\begin{array}{c} {\text{net}}_{i}=\sum_{j=1}^{M}w_{ij}x_{j}+\theta_{i}. \end{array}$$

According to the output formula of the basic neuron, we can find that the output value of the neuron is

(10) $$\begin{array}{c} y_{i}=\phi \left(net_{i}\right)=\phi \left(\sum_{j=1}^{M}w_{ij}x_{j}+\theta_{i}\right). \end{array}$$

By analogy, the input value *net*_*k*_ and output value *o*_*k*_ of the *k*-th node in the hidden layer can be obtained in the following equation:

(11) $$\begin{array}{c} {\text{net}}_{k}=\sum_{i=1}^{q}w_{ij}x_{j}+a_{k}=\sum_{i=1}^{q}w_{ij}\phi \left(\sum_{j=1}^{M}w_{ij}x_{j}+\theta_{i}\right)+a_{k},\\ o_{k}=\Psi \left(net_{k}\right)=\Psi \left(\sum_{i=1}^{q}w_{ij}x_{j}+a_{k}\right)=\Psi \left(\sum_{i=1}^{q}w_{ij}\phi \left(\sum_{j=1}^{M}w_{ij}x_{j}+\theta_{i}\right)+a_{k}\right). \end{array}$$

After determining the input value and output value of each layer of neurons, the corresponding error value can be obtained through the calculation with the real value. The final error value can be obtained by calculating the error value of each neuron and the error value of each layer, respectively. See formula (12) for the specific solution formula.

(12) $$\begin{array}{c} E_{p}=\frac{1}{2}{\sum_{k=1}^{L}\left(T_{k}-o_{k}\right)}^{2}. \end{array}$$

Then, according to the error gradient method, the parameters and thresholds of each layer are continuously adjusted and the final error value is calculated so that the modified parameters and thresholds can minimize the error between the predicted value and the real value. See formulas (13) and (14) for the solution process of the specific modification.

(13) $$\begin{array}{c} \Delta \theta_{i}=-\eta \frac{\partial E}{\partial \theta_{i}}=-\eta \frac{\partial E}{\partial {\text{net}}_{i}}\frac{\partial {\text{net}}_{\text{i}}}{\partial \theta_{i}}=-\eta \frac{\partial E}{\partial y_{i}}\frac{\partial y_{i}}{\partial {\text{net}}_{\text{i}}}\frac{\partial {\text{net}}_{\text{i}}}{\partial \theta_{i}}. \end{array}$$

By simplifying,

(14) Δθ=iη∑p−1P∑k−1LTkp−okp·Ψ′netk·wki·ϕ′neti.

**Figure 5:**
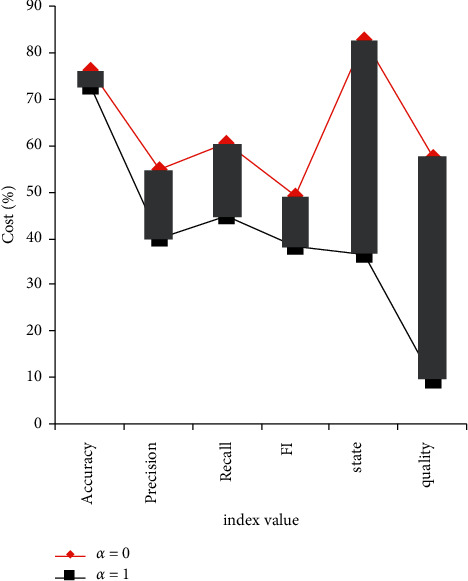
The index value changes under different parameter values.

The variation of error value and accuracy under different parameters is shown in Figure 5.

It can be seen from Figure 5 that the adjusted parameter value has a certain impact on the final result, but the impact is different among different indicators. The parameter value was adjusted from 0 to 1, and the accuracy increased by 5 percentage points, but at the same time, the accuracy showed a downward trend, and the loss value also increased. However, the rise and fall range of each index value is not fixed, but with different parameter values, the impact of adjusting different parameter values is also different. Due to space limitations, this paper will not be repeated one by one. However, we should pay attention to the different effects of parameters in practical training, and we can not blindly pursue high accuracy and ignore other effects.

**Figure 6:**
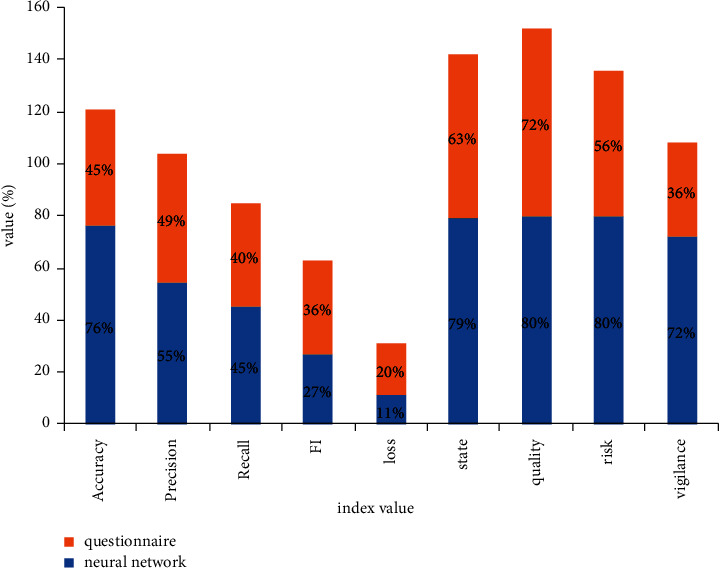
Neural network algorithm is compared with questionnaire survey method.

In order to further verify the accuracy of the neural network algorithm model, we compared it with the traditional survey method, the questionnaire method. We hope to find out the advantages and disadvantages of each method through the neural network model and the comparison between the real results of college students and the results of a questionnaire survey and reuse each other's advantages in their own algorithms.  Figure 6 shows the prediction of the collected objective university mental health information by the neural network, compared with the indicators reflected in the psychological test actually completed by college students.

## 4. Result Analysis and Discussion

**Figure 7:**
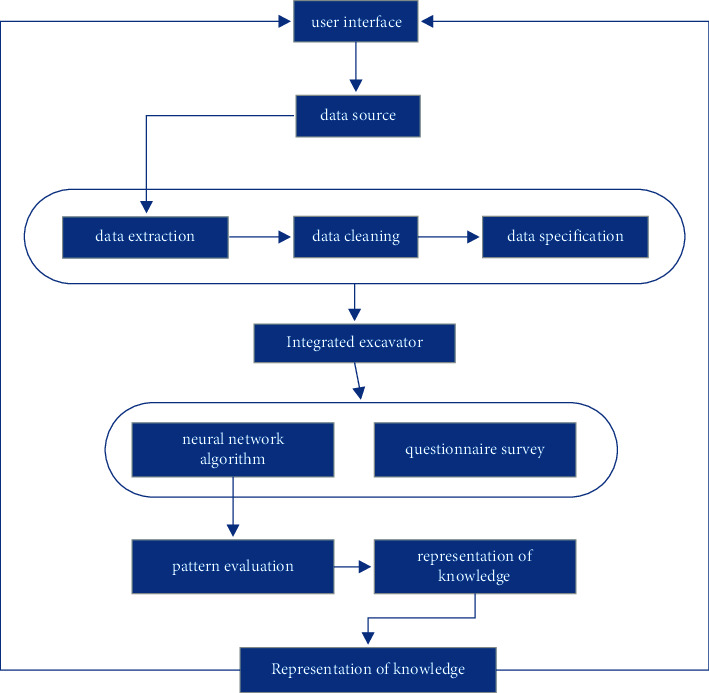
Basic experimental procedure.

When using the model to predict the mental health status of college students, we first need to collect, analyze, and predict the object, that is, the analysis data source. Simple data sources can be derived from the educational administration system. According to previous studies, the mental health status of college students is affected by various factors such as family environment, major, gender, and dormitory relationship. Therefore, we can obtain this kind of information directly. In addition, we need to further obtain the data. Here we use data mining technology to further obtain more information. The study investigated the level of college students' learning and employment in the past two years. In the past two years, more and more people have taken part in China's graduate examination and civil service employment examination. The survey results show that from 2010 to 2020, the number of graduate candidates has increased year by year, but the admission rate has not changed significantly every year. Data mining technology is used to further obtain more information. Data mining technology is a broader category than neural network technology. Neural network technology is one of the commonly used data mining technologies. Therefore, the main flow of this experiment is shown in Figure 7.

Finally, the collected data are put into the neural network model for training. The results showed that the incidence of psychological violence, obsessive-compulsive circumstances, and mental illness had nothing to do with gender. Universities are mainly distributed in cities with a relatively developed economy. Rural students suddenly came to a living environment with a big gap from the past. They need to learn new things and want to adapt to a lifestyle larger than urban students.

There was no significant correlation between College Students' sense of inferiority, dependency map and the incidence of mental diseases and majors. However, there are significant differences in anxiety, depression, and impulsive behavior among students of different majors. At the same time, there are significant regional differences in the expressiveness of urban and rural college students, such as compulsive plot, anxiety plot, social fear, and so on. This performance is the same as we predicted. This shows that students of different majors bear different pressures, so there are significant differences in their mental health. In view of this situation, we can selectively provide special psychological counseling for professional students with high psychological pressure.

**Figure 8:**
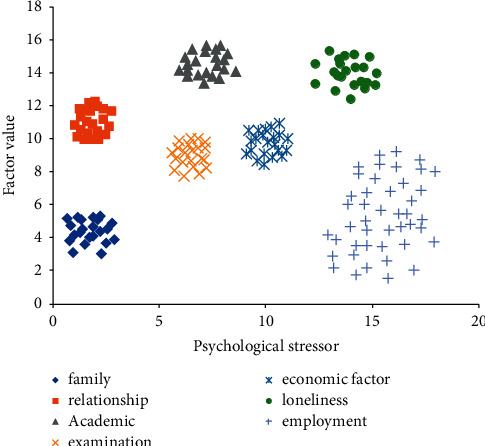
Cluster analysis of stress sources of contemporary college students.

At the same time, there are significant regional differences in the expressiveness of urban and rural college students, such as compulsive plot, anxiety plot, social fear, and so on. This performance is the same as we predicted. In addition, the cluster analysis of psychological stressors of contemporary college students is shown in Figure 8.

From Figure 8, we can see the main sources of the pressure of contemporary college students. The biggest source is employment. China is rich in the labor force. At the same time, with the development of national education in recent years, college students are increasingly facing the problem of being unable to find their favorite job after graduation. At the same time, affected by the epidemic in the past two years, the global economy is in a downturn and the unemployment rate is rising. Contemporary college students often feel strong employment pressure before they leave school. An important manifestation is that in the past two years, more and more people have taken part in the postgraduate examination and civil servant recruitment examination in China.

**Figure 9:**
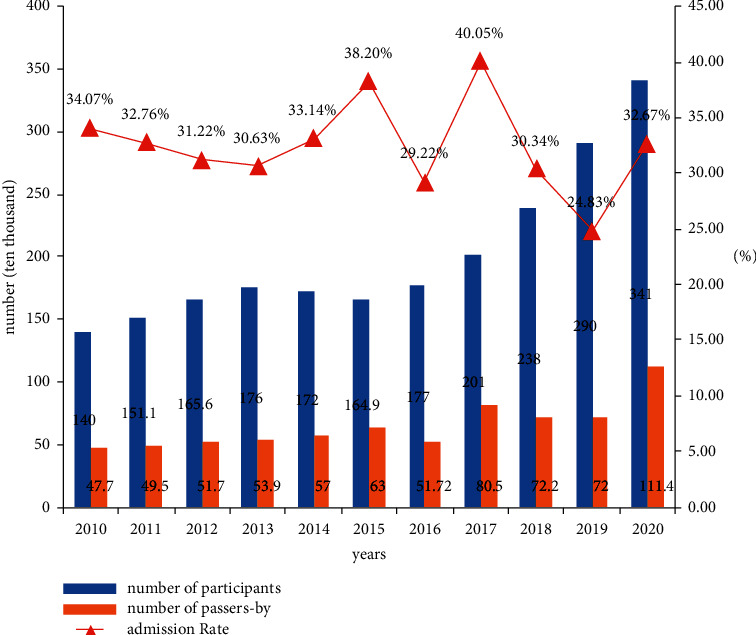
Enrollment of postgraduate students from 2010 to 2020.

In the final analysis, the poor employment environment has led more and more people to reconsider improving their competitiveness or the “iron rice bowl” position of civil servants. This has led to an increase in the pressure of many college students facing the postgraduate entrance examination year by year. The survey results show that from 2010 to 2020, the number of candidates for the postgraduate examination has increased year by year, but there is no significant change in the admission rate every year, which means that more people fail every year (see Figure 9 for the specific results). In the face of these predicted situations, we also need further targeted psychological counseling.

## 5. Conclusion

This paper starts with the worrying mental health of contemporary college students. The computer technology of neural network algorithm is applied to the prediction of contemporary college students' mental health. Data sources are collected by using data mining technology based on a neural network algorithm. Finally, the prediction results are analyzed, and the main psychological stressor factors of contemporary college students are analyzed by cluster analysis. The results show that there is no significant correlation between college students' inferiority complex and dependency map and the incidence of mental diseases and majors. A comprehensive test is conducted on the individual in terms of physical symptoms to understand the students' psychological characteristics and behavior. A psychological evaluation can evaluate students' differences in learning or ability, personality characteristics, relative strengths, and weaknesses, evaluate the development stage that students have reached, determine their relative strengths and weaknesses, and find the reasons for behavior changes. A psychological evaluation can determine the differences among students, predict the possible differences of different individuals in future activities, and speculate the possibility of students' future success in a certain field.

## Data Availability

The data used to support the findings of this study are available from the author upon request.
